# Phthalates and Perfluorooctanesulfonic Acid in Human Amniotic Fluid: Temporal Trends and Timing of Amniocentesis in Pregnancy

**DOI:** 10.1289/ehp.1104522

**Published:** 2012-03-07

**Authors:** Morten Søndergaard Jensen, Bent Nørgaard-Pedersen, Gunnar Toft, David M. Hougaard, Jens Peter Bonde, Arieh Cohen, Ane Marie Thulstrup, Richard Ivell, Ravinder Anand-Ivell, Christian H. Lindh, Bo A.G. Jönsson

**Affiliations:** 1 Danish Ramazzini Centre, Department of Occupational Medicine, Aarhus University Hospital, Aarhus, Denmark; 2 Perinatal Epidemiology Research Unit, Department of Pediatrics, Aarhus University Hospital, Skejby, Denmark; 3 Section of Neonatal Screening and Hormones, Department of Clinical Biochemistry and Immunology, Statens Serum Institut, Copenhagen, Denmark; 4 Department of Occupational and Environmental Medicine, Bispebjerg Hospital, University of Copenhagen, Copenhagen, Denmark; 5 Department of Reproductive Endocrinology, and; 6 Department of Reproductive Cell Biology, Leibniz Institute for Farm Animal Biology, Dummerstorf, Germany; 7 Division of Occupational and Environmental Medicine, Department of Laboratory Medicine, Lund University, Lund, Sweden

**Keywords:** amniocentesis, amniotic fluid, biobank, biomonitoring, perfluorinated compounds, phthalates, pregnancy, temporal trend

## Abstract

Background: Measures of prenatal environmental exposures are important, and amniotic fluid levels may directly reflect fetal exposures during hypothesized windows of vulnerability.

Objectives: We aimed to detect various phthalate metabolites and perfluorooctanesulfonic acid (PFOS) in human amniotic fluid, to study temporal exposure trends, and to estimate potential associations with gestational week of amniocentesis and maternal age and parity at amniocentesis.

Methods: We studied 300 randomly selected second-trimester amniotic fluid samples from a Danish pregnancy-screening biobank covering 1980 through 1996. We used only samples from male offspring pregnancies. We assayed the environmental pollutants by liquid chromatography/triple quadrupole mass spectrometry and analyzed data using generalized linear regression models.

Results: We detected the di(2-ethylhexyl) phthalate (DEHP) metabolite mono(2-ethyl-5-carboxypentyl) phthalate (5cx-MEPP) at a median concentration of 0.27 ng/mL [interquartile range (IQR): 0.20–0.37 ng/mL], the diisononyl phthalate (DiNP) metabolite mono(4-methyl-7-carboxyheptyl) phthalate (7cx-MMeHP) at 0.07 ng/mL (IQR: 0.05–0.11 ng/mL), and PFOS at 1.1 ng/mL (IQR: 0.66–1.60 ng/mL). An increase of 1 calendar year was associated with 3.5% lower [95% confidence interval (CI): –4.8%, –2.1%] 5cx-MEPP levels and with 7.1% higher (95% CI: 5.3%, 9.0%) 7cx-MMeHP levels. For each later gestational week of amniocentesis, 5cx-MEPP was 9.9% higher (95% CI: 4.8%, 15.2%), 7cx-MMeHP was 8.6% higher (95: CI: 2.7%, 14.9%), and PFOS was 9.4% higher (95: CI: 3.3%, 15.9%). We observed no associations with maternal age or parity.

Conclusions: Measured metabolite levels appeared to parallel decreasing DEHP exposure and increasing DiNP exposure during the study period. The environmental pollutant levels were positively associated with later gestational age at amniocentesis during pregnancy weeks 12–22.

Prenatal exposures to phthalates and perfluorinated compounds (PFCs) are suspected to cause immediate and long-term adverse health effects in the offspring (Jurewicz and Hanke 2011; Lau et al. 2007; Matsumoto et al. 2008; Olsen et al. 2009). Exposures are ubiquitous and well documented in pregnant women (Adibi et al. 2003; Fromme et al. 2009; Woodruff et al. 2011; Yan et al. 2009). Direct fetal exposure, however, is less easily determined (Arbuckle 2010). Measurements in blood or urine from pregnant women may serve as proxies, but correlations with measures in fetal compartments can be low (Huang et al. 2009; Latini et al. 2003; Wittassek et al. 2009). Placental transfer of phthalates and PFCs has been documented in rodents (Calafat et al. 2006; Yu et al. 2009) and humans (Inoue et al. 2004; Latini et al. 2003). Human *ex vivo* placental perfusion studies generally suggested lower phthalate levels in the fetal circulation than in the maternal circulation (Mose et al. 2007a, 2007b). Paired measurements have suggested either identical or lower phthalate levels in fetal cord blood compared with maternal blood (Chen et al. 2008; Latini et al. 2003; Yan et al. 2009; Zhang et al. 2009) and generally lower fetal levels of PFCs (Fromme et al. 2009, 2010; Hanssen et al. 2010; Monroy et al. 2008). In practice, cord blood is obtainable only at birth, but the expected vulnerable time window for many health outcomes is often earlier in pregnancy (e.g., during organogenesis).

Amniotic fluid offers another possible fetal matrix, but samples are most often available only from amniocentesis indicated by advanced maternal age or by prenatal diagnostics of chromosomal abnormalities and severe malformations. Another challenge, which is not unique to amniotic fluid (Sibai and Frangieh 1995), is the dynamic changes in amniotic fluid volume and composition throughout pregnancy (Benzie et al. 1974; Brace 1997; Modena and Fieni 2004), which complicate interpretation of measured pollutant concentrations. One major advantage, however, is that amniocentesis usually is performed within the second trimester, which for many outcomes may be closer to the expected vulnerable period. This may be more important for the short-lived phthalate metabolites than for the persisting PFCs (Lau et al. 2007; Wittassek and Angerer 2008). Only three studies comprising a total of 129 samples have reported phthalate metabolites in human amniotic fluid (Huang et al. 2009; Silva et al. 2004; Wittassek et al. 2009), and to the best of our knowledge, no published studies have reported measures of PFCs in human amniotic fluid. The scarce nature of such measurements limits our knowledge about exposure levels, kinetics, metabolism and elimination. Further, none of the previous studies have been able to investigate temporal exposure trends or potential associations with important covariates such as gestational age, maternal parity, and maternal age at amniocentesis.

We aimed to describe levels of phthalate metabolites and perfluorooctanesulfonic acid (PFOS) in human amniotic fluid samples from a Danish biobank. In addition, we investigated temporal trends in exposure levels, as well as potential associations with gestational age, maternal age, and parity at amniocentesis.

## Methods

*Study population and amniotic fluid samples.* We used amniotic fluid samples from a Danish biobank maintained at the State Serum Institute in Copenhagen. The biobank holds samples from a pregnancy screening registry including both amniotic fluid and maternal serum samples from > 100,000 pregnancies covering the period 1979 through 2004 (amniotic fluid samples 1980–1996). The amniotic fluid samples were centrifuged before routine diagnostic analyses and the supernatants were kept frozen at –20°C until the present analyses were carried out. The samples were from Sealand (Copenhagen hospitals and Hillerød Hospital) and from Southern Jutland (Sønderborg and Kolding Hospitals). The main indication for amniocentesis was age ≥ 35 years, but some samples were from women with increased risk of severe malformations or Down syndrome based on results from maternal serum analyses.

Each mother’s personal identification number (unique to each Danish citizen) was recorded in the pregnancy screening registry and also identified her amniotic fluid sample. We used the unique identifiers of all women in the pregnancy screening registry to obtain obstetric data on all their pregnancies from the Danish Medical Birth Registry (Knudsen and Olsen 1998), including gestational age at birth, singleton or multiple birth, maternal parity, and birth weight and Apgar score of the infant. In addition, we used the unique identifiers in the Danish Civil Registration System to identify each mother’s children (Pedersen et al. 2006). We included only live-born boys because this study was part of a larger study on male urogenital anomalies. To verify that the amniotic fluid sample and the identified boy originated from the same pregnancy, we selected only those where the amniocentesis had been performed between 10 and 30 weeks from the estimated date of conception, defined by subtracting the gestational age at birth from the date of birth. We then followed the identified boys in the Danish National Patient Registry until November 2008 to obtain records of urogenital anomalies and other congenital malformations, including chromosomal abnormalities [*International Classification of Diseases, 8th Revision* (ICD-8; World Health Organization 1969), codes 74000–75999; ICD-10 (*10th Revision*; World Health Organization 1993) codes Q00–Q99] (Andersen et al. 1999). We selected a random sample for this study among all live-born singleton boys born to women registered in the pregnancy-screening registry and with complete obstetric data.

The gestational age at birth was determined by last menstrual period and corrected by ultrasound according to local guidelines if necessary. Obstetric ultrasound became increasingly available in Denmark during the study period and was performed on approximately 93% of all pregnant women by 1995 (Jørgensen 1999). We calculated gestational week of amniocentesis as the distance between the estimated date of conception (defined above) and the date of amniocentesis.

The Danish Regional Ethics Committee, the Danish National Board of Health, and the Danish Data Protection Agency approved the study. The use of the biobank for research purposes has been approved, and additional informed consent from the study subjects for this specific project was neither recommended nor required.

*Chemical analysis.* We assayed the di(2-ethylhexyl) phthalate (DEHP) metabolites mono(2-ethyl-5-hydroxylhexyl) phthalate (5OH-MEHP), mono(2-ethyl-5-oxohexyl) phthalate (5oxo-MEHP), and mono(2-ethyl-5-carboxypentyl) phthalate (5cx-MEPP) and the diisononyl phthalate (DiNP) metabolites mono(4-methyl-7-hydroxyoctyl) phthalate (7OH-MMeOP), mono(4-methyl-7-oxooctyl) phthalate (7oxo-MMeOP), and mono(4-methyl-7-carboxyheptyl) phthalate (7cx-MMeHP), as well as PFOS and cotinine, in the amniotic fluid samples during the summer and fall of 2010. We detected 5cx-MEPP, 7cx-MMeHP, PFOS, and cotinine, and the method had good reproducibility as determined from duplicate samples analyzed at different days. Coefficients of variation were between 9% and 16%, and we achieved rather low limits of detection (LOD), which were determined as the concentrations corresponding to three times the standard deviation of the responses in chemical blanks [for more details, see Supplemental Material, Table S1 (http://dx.doi.org/10.1289/ehp.1104522)]. We monitored the quality by analyzing chemical blanks and in-house quality-control samples in all batches.

We added 10 μL 1 M ammonia acetate and 10 μL glucoronidase from *Escherichia coli* to aliquots of 100 μL amniotic fluid. After mixing, we incubated the samples at 37°C for 90 min. We prepared standards from amniotic fluid added with known amounts of phthalate metabolites, PFOS, and cotinine in 25 μL of a 50:50 solution of water and acetonitrile. We added 25 μL of the same solution but without the compounds to the samples, and then added 25-μL aliquots of a 50:50 water:acetonitrile solution of ^13^C- and ^2^H-labeled internal standards for all analyzed compounds. We precipitated the proteins by adding 150 μL acetonitrile and vigorously shaking for 30 min, and afterward we centrifuged the samples and transferred them to autosampler vials.

We analyzed the samples using a liquid chromatograph (LC; model UFLCXR, Shimadzu Corp., Kyoto, Japan). In the analysis of the phthalates, we injected aliquots of 5 μL on a C18 column (4 μm, 2.1 mm inner diameter × 50 mm GENISIS; Grace Vydac, Hesperia, CA, USA). The mobile phases consisted of 0.08% formic acid in water and acetonitrile. The separation started at 20% acetonitrile, followed by a linear gradient of acetonitrile to 75% in 3 min. We washed the column by 95% acetonitrile and then equilibrated in 20% acetonitrile during 2 min. In the analysis of PFOS, we injected aliquots of 3 μL on a C18 column (4 μm, 2.1 mm inner diameter × 50 mm GENISIS). The mobile phases consisted of 0.1% ammonia in water and acetonitrile. The separation started with a 5-min isocratic step at 30% acetonitrile, followed by a linear gradient of acetonitrile to 95% in 2 min. We then equilibrated the column in 30% acetonitrile for 2 min. In the analysis of cotinine, we injected aliquots of 3 μL on a C18 Hypersil GOLD column (5 μm, 3 mm inner diameter × 150 mm; Thermo Scientific, Waltham, MA, USA). The mobile phases consisted of 0.1% ammonia in water and acetonitrile. The separation started with a 1-min isocratic step at 25% acetonitrile, followed by a linear gradient of acetonitrile to 95% for 3 min. We then equilibrated the column in 25% acetonitrile for 2 min. The LC was connected to a hybrid triple quadrupole linear ion trap tandem mass spectrometer (LC/MS/MS) equipped with a turbo ion spray source (QTRAP 5500; AB Sciex, Foster City, CA, USA). For technical parameters and for sample chromatograms, see Supplemental Material, Table S2 and Figures S1, S2 (http://dx.doi.org/10.1289/ehp.1104522).

Recent participation in interlaboratory urine sample control programs of several phthalate metabolites, including 5cx-MEPP and 7cx-MMeHP, and of cotinine yielded results good enough for the laboratory to become a reference laboratory in a large European biomonitoring program [Consortium to Perform Human Biomonitoring on a European Scale (COPHES) 2012]. Furthermore, the laboratory’s analyses of PFOS in serum and of 5cx-MEPP and cotinine in urine are part of the Round Robin intercomparison program (H. Drexler, Institute and Out-Patient Clinic for Occupational, Social and Environmental Medicine, University of Erlangen-Nuremberg, Germany), with results within the tolerance limits.

*Statistical analysis.* We compared pregnancy characteristics by amniotic fluid sample availability to evaluate potential selection bias. We tabulated proportions or means and used Pearson’s chi-square test for categorical variables and the *t*-test for continuous variables when comparing the groups.

Less than 5% of the values from the chemical assays of the four detected environmental pollutants were below the LOD, and they were imputed with a random value between LOD and LOD/2. For each pollutant we present the LOD, the proportion of samples above LOD and the 10th, 25th, 50th, 75th, and 100th percentiles.

We estimated potential associations of calendar year, gestational age, and maternal age and parity at amniocentesis with each pollutant in generalized linear regression models with squared terms of the numerical explanatory variables to estimate deviations from linearity. We transformed the environmental pollutant measures by the natural logarithm (ln) to normalize the distribution of residuals, and we consequently exponentiated the differences estimated on the ln scale to obtain ratios of the medians. We present these ratios as crude and adjusted percent change per unit of the explanatory variable with 95% confidence intervals (CIs). In addition, we present associations with the numerical explanatory variables as scatter plots with unadjusted linear regression lines. We made mutual adjustments for calendar year of amniocentesis (1980–1984, 1985–1990, 1991–1996), gestational age at amniocentesis (< 15, 15–17, ≥ 17 weeks), maternal age at amniocentesis (< 30, 30–34, ≥ 35 years), maternal smoking estimated by cotinine levels [“nonsmoker” < 25, “passive smoker” 25–85, “active smoker” ≥ 85 ng/mL amniotic fluid (Jauniaux et al. 1999)], and maternal parity (0, 1, ≥ 2 prior births). We estimated correlation coefficients between the ln-transformed environmental pollutants with *p*-values from Spearman rank correlations. All *p*-values were estimated by two-tailed tests, and we considered an α-level of 0.05 statistically significant. Statistical analyses were performed using STATA software (version 11; StataCorp, College Station, TX, USA).

## Results

**Figure 1:**
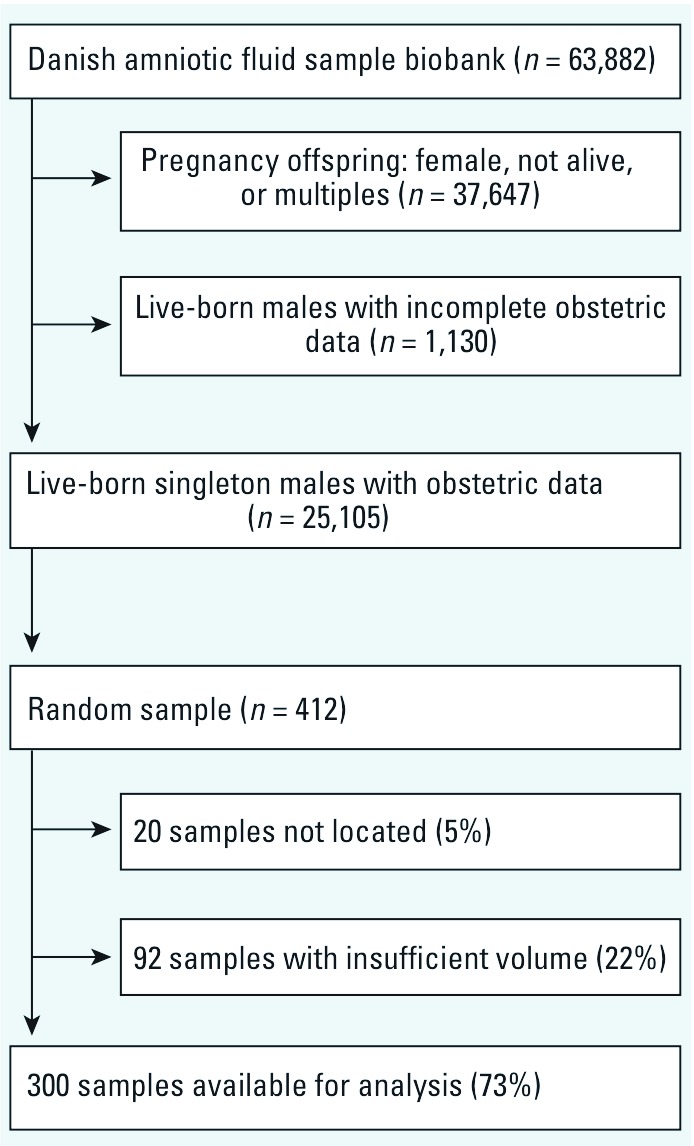
Study population and amniotic fluid samples, Denmark, 1980–1996.

The pregnancy screening registry had records of 63,882 amniotic fluid samples (Figure 1). After restriction to live-born singleton boys with complete obstetric data, 25,105 pregnancies were eligible. We selected a random sample of 412 for this study, of which 20 amniotic fluid samples were not located in the biobank and 92 samples had insufficient volume, leaving 300 samples (73%) available for chemical analyses.

We compared pregnancy characteristics by availability of amniotic fluid samples (Table 1). Mothers with unavailable samples were older and more likely to have had their amniocentesis between 1985 and 1990. Differences by availability were indicated for maternal parity and prevalence of congenital malformations. Other characteristics, such as gestational week of amniocentesis, boys’ Apgar score, gestational age at birth, and birth weight, did not differ between the two groups.

**Table 1:** Pregnancy and offspring characteristics of 412 randomly selected amniotic fluid samples, by availability, Denmark, 1980–1996.

Amniotic fluid samples (n = 412)		
Characteristic	Available (n = 300)	Unavailable (n = 112)	p-Value^a^
Maternal age at birth [years (%)]						0.02
< 30		30		21		
30–34		24		17		
≥ 35		46		62		
Primiparous (%)		35		28		0.14
Year of amniocentesis (%)						< 0.001
1980–1984		33		20		
1985–1990		34		60		
1991–1996		33		20		
Gestational week of amniocentesis (%)						0.78
< 15		27		23		
15–17		57		60		
≥ 17		16		17		
Congenital malformations in boysb (%)		6		11		0.13
Apgar score ≥ 9 (%)		85		84		0.88
Gestational age at birth [weeks (mean ± SD)]		39.6 ± 1.5		39.5 ± 1.8		0.79
Birth weight [g (mean ± SD)]		3,535 ± 561		3,544 ± 664		0.89
aCategorical variables were tested with Pearson’s chi-square test, and continuous variables with the t-test. bCryptorchidism and hypospadias were not included.						

The two detected phthalate metabolites, PFOS, and cotinine were all assayed with > 95% above the LOD (Table 2). Medians were 0.27 ng/mL [interquartile range (IQR): 0.20–0.37 ng/mL] for the DEHP metabolite 5cx-MEPP, 0.07 ng/mL (IQR: 0.05–0.11 ng/mL) for the DiNP metabolite 7cx-MMeHP, 1.1 ng/mL (IQR: 0.66–1.60 ng/mL) for PFOS, and 2.2 ng/mL (IQR: 0.60–144 ng/mL) for cotinine. We estimated a correlation coefficient of 0.16 (*p* < 0.01) between 5cx-MEPP and 7cx-MMeHP, 0.17 (*p* = 0.03) between 7cx-MMeHP and PFOS, and 0.18 (*p* < 0.01) between 5cx-MEPP and PFOS. Maternal smoking (nonsmoker, passive smoker, active smoker) was not associated with gestational age at amniocentesis (< 15, 15–17, ≥ 17 weeks) in a 3 × 3 contingency table (Pearson chi-square *p* = 0.69).

**Table 2:** Amniotic fluid concentrations of detected phthalate metabolites, PFOS and cotinine, Denmark, 1980–1996 (n = 300).

Environmental pollutant	Percentile (ng/mL)										
	LOD (ng/mL)	n (%) ≥ LOD	10th	25th	50th	75th	90th	Maximum
5cx-MEPP		0.05		296 (99)		0.16		0.20		0.27		0.37		0.51		2.3
7cx-MMeHP		0.02		288 (96)		0.02		0.05		0.07		0.11		0.15		0.91
PFOS		0.2		295 (98)		0.43		0.66		1.1		1.6		2.0		4.5
Cotinine		0.2		293 (98)		0.30		0.60		2.2		144		263		531


**Figure 2:**
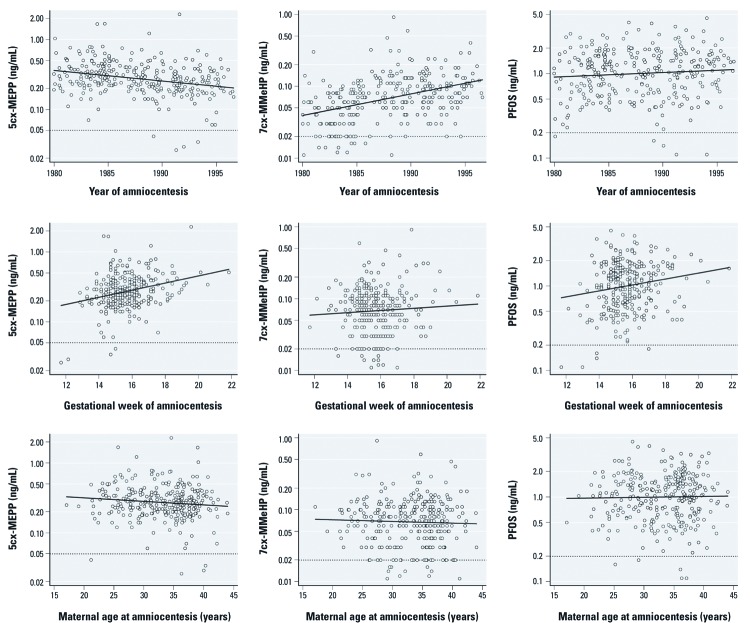
Amniotic fluid 5cx-MEPP (left column), 7cx-MMeHP (middle column) and PFOS (right column) according to year of amniocentesis (top row), gestational week of amniocentesis (middle row), and maternal age at amniocentesis (bottom row): Denmark, 1980–1996. The *y*-axis scale is natural logarithm (ln). Solid lines are unadjusted linear regressions; dotted lines are LODs for each environmental pollutant.

After adjustment, an increase of 1 calendar year was associated with 3.5% lower 5cx-MEPP levels (95% CI: –4.8%, –2.1%), with 7.1% higher 7cx-MMeHP levels (95% CI: 5.3%, 9.0%), and with 1.5% higher PFOS levels (95% CI: –0.3%, 3.2%) (Figure 2, top row; Table 3). 5cx-MEPP, 7cx-MMeHP, and PFOS levels were approximately 8–10% higher for each later gestational week of amniocentesis (Figure 2, middle row; Table 3). These trends were slightly stronger for all pollutants in the most recent years (1991–1996; data not shown). We observed statistically significant deviations from linearity for two associations: The association between year of amniocentesis and 7cx-MMeHP had a very slight curvature that would not invalidate conclusions from a linear model; the association between gestational week of amniocentesis and PFOS had a stronger curvature that was driven by the five imputed values below the LOD [the nonlinearity was removed after exclusion of these observations; for details on these nonlinearities, see Supplemental Material, Figures S3 and S4 (http://dx.doi.org/10.1289/ehp.1104522)]. None of the environmental pollutants were associated with maternal age (Figure 2, bottom row; Table 3) or consistently associated with maternal parity (Table 4).

**Table 3:** Percent change (95% CI) in amniotic fluid environmental pollutant concentration by one unit increase in calendar year of amniocentesis (1980–1996), gestational week of amniocentesis (12–22), and maternal age at amniocentesis (17–44 years).

Percent change^a^										
Environmental pollutant	Per calendar year			Per gestational week			Per year of maternal age		
	Crude	Adjusted (95% CI)b	Crude	Adjusted (95% CI)c	Crude	Adjusted (95% CI)d
5cx-MEPP		–3.4		–3.5 (–4.8, –2.1)		12.5		9.9 (4.8, 15.2)		–1.1		–0.6 (–1.9, 0.8)
7cx-MMeHP		7.1		7.1 (5.3, 9.0)		3.5		8.6 (2.7, 14.9)		–0.6		0.5 (–1.0, 2.1)
PFOS		1.1		1.5 (–0.3, 3.2)		8.4		9.4 (3.3, 15.9)		0.1		0.4 (–1.2, 2.0)
aThe ratio of medians estimated by linear regression with the pollutant concentration on the ln scale. bAdjusted for maternal smoking, parity, gestational age at amniocentesis, and maternal age. cAdjusted for maternal smoking, parity, year of amniocentesis, and maternal age. dAdjusted for maternal smoking, parity, year of amniocentesis, and gestational age at amniocentesis.												

**Table 4:** Environmental pollutant concentration in amniotic fluid according to maternal parity at amniocentesis, Denmark, 1980–1996.

5cx-MEPP							7cx-MMeHP							PFOS						
Parity	n	Mean (ng/mL)	Median (ng/mL)	Percent change (95% CI)a	p-Valueb	Mean (ng/mL)	Median (ng/mL)	Percent change (95% CI)a	p-Valueb	Mean (ng/mL)	Median (ng/mL)	Percent change (95% CI)a	p-Valueb
0		106		0.32		0.26						0.09		0.07						1.21		1.08				
1		93		0.35		0.26		9.4 (–6.5, 28.1)		0.82		0.10		0.08		21.8 (1.2, 46.6)		0.06		1.23		1.03		–2.5 (–19.4, 17.9)		0.76
≥ 2		101		0.30		0.27		–4.3 (–18.8, 12.9)		0.64		0.08		0.06		–5.6 (–22.3, 14.5)		0.17		1.18		1.09		–2.2 (–19.8, 19.3)		0.84
aThe ratio of medians estimated by linear regression with the pollutant concentration on the ln scale and adjusted for year of amniocentesis, gestational age at amniocentesis, maternal age, and maternal smoking. Reference group is nullipara. bWilcoxon rank-sum test with nullipara as reference group.																										

## Discussion

We studied the concentrations of phthalate metabolites and PFOS in the largest set of human amniotic fluid samples reported so far. We were able to detect one DEHP metabolite (5cx-MEPP), one DiNP metabolite (7cx-MMeHP), and PFOS. Concentrations of these environmental pollutants were low, and correlations between them were inconsequential. We observed metabolite levels suggesting decreasing DEHP exposure and increasing DiNP exposure during the study period of 1980–1996.

Our cross-sectional data suggested higher levels of all three environmental pollutants for each later gestational week of amniocentesis during weeks 12–22. This trend of higher concentrations in later weeks coincides with increasing fetal urine excretion and amniotic fluid volume expansion (Gilbert and Brace 1993; Lotgering and Wallenburg 1986). The phthalate metabolites and PFCs are to some extent protein bound, and the total protein concentration in amniotic fluid also increases during weeks 12–22 (Griffiths et al. 1988; Jauniaux et al. 1994; Jones et al. 2003; Queenan 1978). Although these physiological changes might account for part of the associations, it is probably premature to fully explain these identical trends in various environmental pollutants with different half-lives (Lau et al. 2007; Wittassek and Angerer 2008). However, the findings do underscore the importance of including gestational age at amniocentesis in statistical analyses of environmental pollutant levels in amniotic fluid.

Gestational age determination has inherent limitations, but any random misclassification would most likely attenuate the observed positive associations with levels of the environmental pollutants. Theoretically, the associations could be produced by mothers with high environmental pollutant levels (or lifestyles associated with high levels) having their amniocentesis later, due to, for example, later recognition of pregnancy. We observed no association between amniotic fluid cotinine levels (maternal smoking) and gestational week at amniocentesis, which is reassuring regarding this potential bias mechanism. Another systematic error could occur if high levels of the environmental pollutants are associated with irregular menstrual cycle (Fei et al. 2009). This would only affect gestational age determined by last menstrual period, which was most frequent in the first part of the study period. The association with gestational week of amniocentesis was slightly stronger for all pollutants in the most recent years (1991–1996). During this period, approximately 90% of Danish pregnant women had an obstetric ultrasound free of charge (Jørgensen 1999), which would tend to reduce this possible bias (if present). It would, however, require replication by paired within-person measurements during pregnancy to draw definitive conclusions of a genuine increase in pollutant levels during weeks 12–22.

Several DEHP and DiNP metabolites have been detected in human amniotic fluid from Taiwan during 2005–2006 (Huang et al. 2009) and from the United States (Silva et al. 2004) and Germany (Wittassek et al. 2009) during undescribed time periods (Table 5). Mono(2-ethylhexyl) phthalate (MEHP) levels were reported > 10 times higher in Taiwan (22.1 ng/mL; Huang et al. 2009) than in Germany (1.6 ng/mL; Wittasek et al. 2009). Human placental perfusion studies have shown no or very low transfer of MEHP to fetal compartments (Mose et al. 2007a, 2007b), and whether the reported amniotic fluid MEHP levels reflect true geographical differences in exposure, varying laboratory methods, or contamination remains a puzzle. Some measures were taken to avoid contamination in the Taiwanese study (Huang et al. 2009), but amniotic fluid may have lipase or esterase activity that hydrolyses phthalate diesters to monoesters. Thus, if monoesters are the analytic goal, samples must be acidified immediately after amniocentesis (which was not done) to avoid enzyme activity. Unlike with monoesters, the secondary metabolites are unlikely subjects of contamination (Wittassek and Angerer 2008). Among secondary DEHP metabolites the carboxy-metabolites 5cx-MEPP and mono(2-carboxy-hexyl) phthalate (2cx-MMHP) have been most readily detected (Table 5). 5cx-MEPP was measured within the same range of magnitude in Germany (Wittasek et al. 2009) and Denmark (present study). Among secondary DiNP metabolites, the German study (Wittasek et al. 2009) detected 7cx-MMeHP in only 1 of 11 samples but at a rather high level of 4.9 ng/mL. We detected 7cx-MMeHP in 96% of the samples in our study with a lower LOD. 7cx-MMeHP and 5cx-MEPP are major urinary metabolites of DiNP and DEHP respectively (Jönsson BAG, unpublished data; Silva et al. 2006). Hence, it is not surprising that these metabolites were also detected in amniotic fluid and that others were below LODs. In addition, 5cx-MEPP has a longer half-life in urine than do 5OH-MEHP and 5oxo-MEHP (Heudorf et al. 2007), thus making 5cx-MEPP a more stable urine biomarker of DEHP exposure. Based on this, 5cx-MEPP and 7cx-MMeHP may also likely be the most stable DEHP and DiNP biomarkers in amniotic fluid if elimination patterns compare with urine. DiNP is a widely used modern phthalate that has to some extent replaced DEHP (Wittassek et al. 2007). For this reason, it is important to develop methods to measure DiNP metabolites for exposure biomonitoring and etiological research purposes. Furthermore, this study provides the first published detection of PFOS, another important environmental pollutant, in human amniotic fluid.

**Table 5:** DEHP and DiNP metabolites currently detected by MS in human amniotic fluid.

Metabolite	Studya	Detected	LOD (ng/mL)	Median (ng/mL)
DEHP								
MEHP		1		(+)		0.86		< LOD
		2		+		0.90		22.10
		3		+		0.15		1.60
5OH-MEHP		1		–		—		—
		3		(+)		0.15		< LOD
		4		–		0.10		—
5oxo-MEHP		1		–		—		—
		3		(+)		0.15		< LOD
		4		–		0.03		—
5cx-MEPP		3		+		0.15		0.53
		4		+		0.05		0.27
2cx-MMHP		3		+		0.15		0.64
DiNP								
Monoisononyl phthalate		1		–		—		—
7OH-MMeOP		3		–		0.15		—
		4		–		0.01		—
7oxo-MMeOP		3		–		0.15		—
		4		–		0.02		—
7cx-MMeHP		3		(–)b		0.15		—
		4		+		0.02		0.07
Symbols: – not detected, (+) poorly detected (median below LOD), + detected, — not available. aStudies: 1, Silva et al. (2004), United States; 2, Huang et al. (2009), Taiwan; 3, Wittassek et al. (2009), Germany; 4, present study, Denmark. b7cx-MMeHP was detected in only 1 of 11 samples.								

A few studies reported temporal trends in exposures to phthalates and PFCs in Denmark’s neighbor countries. A study from Germany estimated daily phthalate intake based on 24-hr urinary metabolite excretion in 634 students 20–29 years of age during 1988–2003 (Wittassek et al. 2007). Data strongly suggested a decrease in DEHP exposure and an increase in DiNP exposure over time, which is exactly what we observed during the years 1980–1996 (Figure 2, top row). PFOS exposure trends were considerably more heterogeneous; a study from Norway using pooled serum samples reported an increase in young females’ PFOS levels from 2.5 ng/mL in 1976 to 25 ng/mL in 1998 (Haug et al. 2009), but during almost the same period we observed a very limited increase, with CIs including no change (Figure 2, top row). A small study from Germany observed an increase in young students’ plasma PFOS levels from 10 ng/mL in 1977 to 26 ng/mL in 1990 (based on 18 samples) (Wilhelm et al. 2009). However, a decline in PFOS levels in German students was reported from 1985 to 2004, partly covering the same period (Wiesmuller et al. 2007). Hence, data on PFOS time trends remain rather inconsistent in these comparable populations.

We used samples from a large pregnancy-screening biobank. The main indication for amniocentesis was ≥ 35 years of age (46% of mothers; Table 1), but samples were also from women with increased risk of severe malformations or Down syndrome based on results from maternal serum analyses. Selective participation in the pregnancy-screening program remains a theoretic possibility, but the screening was offered to all eligible women by their family doctor or obstetrician and free of charge. We selected a random sample from the database, but approximately 27% of that sample was not available because of missing amniotic fluid samples or insufficient volume (Figure 1). Several characteristics, such as gestational week of amniocentesis, boys’ Apgar score, gestational age at birth, and birth weight, did not differ by availability. Available samples were evenly distributed across calendar years, whereas unavailable samples were more often from the middle of the study period (Table 1). Mothers with available samples were younger, and we speculate that the obtained volume by amniocentesis or the analytical volume needed may differ between routine screening assays (age ≥ 35 years) and assays because of suspected pathology (age more frequently < 35 years). It remains difficult, however, to understand why selection by remaining volume would bias our findings.

We performed the chemical analyses blinded to all other information, and we randomized the samples before analyses. The levels of environmental pollutants were very low, especially for the phthalate metabolites. We used a newly developed, simple, and rapid workup method for the determinations, requiring only very low sample volume and using the latest generation of highly sensitive LC/MS/MS equipment. The achieved LODs were generally much lower than for previously described methods (Huang et al. 2009; Silva et al. 2004; Wittassek et al. 2009), although the imprecision might increase at the lowest levels. The choice of imputation procedure for the < 5% of samples below LOD is not expected to impact our findings.

Our data were based only on male offspring pregnancies and might not be representative for female offspring pregnancies if they have different fetal or maternal metabolism or excretion (Kudo and Kawashima 2003; Lau et al. 2004). However, recent evidence suggests no sex differences in renal excretion of PFCs in humans (Harada et al. 2005), and comparable levels in both sexers have been observed for several phthalate metabolites (Huang et al. 2009).

## Conclusion

We detected one DEHP metabolite, one DiNP metabolite, and PFOS in human amniotic fluid. Other assayed phthalate metabolites were below LODs. Measured metabolite levels appeared to parallel decreasing DEHP exposure and increasing DiNP exposure during the study period of 1980–1996. Further, we observed positive associations between levels of the three measured environmental pollutants and gestational age at amniocentesis during weeks 12–22. Levels of the pollutants in amniotic fluid were not associated with maternal age or parity.

## Supplemental Material

(201 KB) PDF
