# Supplementary material for: Phthalates and Perfluorooctanesulfonic Acid in Human Amniotic Fluid: Temporal Trends and Timing of Amniocentesis in Pregnancy
Source: Environ Health Perspect. 2012 Mar 7;120(6):897–903. doi: 10.1289/ehp.1104522 (PMC3385442; doi:10.1289/ehp.1104522)
Supplement: (201 KB) PDF [file ehp.1104522.s001.pdf]

SUPPLEMENTAL MATERIAL

Phthalates and Perfluorooctanesulfonic Acid in Human Amniotic Fluid: Temporal Trends and Timing of Amniocentesis in Pregnancy

Morten Søndergaard Jensen, Bent Nørgaard-Pedersen, Gunnar Toft, David M. Hougaard, Jens Peter Bonde, Arieh Cohen, Ane Marie Thulstrup, Richard Ivell, Ravinder Anand-Ivell, Christian H. Lindh and Bo A.G. Jönsson

Table of contents

Table S1 (Details on the environmental pollutants assayed)…………………………………………… Page 2
Table S2 (Summary of technical parameters)……………………………………………………………. Page 3
Figure S1 (Sample chromatograms—Phthalates)…………………………………………………...Page 4
Figure S2 (Sample chromatogram—PFOS)………………………………………………………….....Page 5
Figure S3 (Non-linearity: 7cx-MMeHP versus year of amniocentesis)…………………………………Page 6
Figure S4 (Non-linearity: PFOS versus gestational week of amniocentesis)………....................Page 7

Table S1.
Environmental pollutants assayed in 300 human amniotic fluid samples by liquid chromatography mass spectrometry (LC/MS/MS), Denmark 1980-1996

Environmental pollutant	Metabolite	LOD (ng/ml)	Detected	CV (%)	Level (ng/ml)
Di(2-ethylhexyl) phthalate [DEHP]	Mono(2-ethyl-5-carboxypentyl) phthalate [5cx-MEPP]	0.05	Yes	16	1
	Mono(2-ethyl-5-hydroxyhexyl) phthalate [5OH-MEHP]	0.10	No		
	Mono(2-ethyl-5-oxohexyl) phthalate [5oxo-MEHP]	0.03	No		
Diisononyl phthalate [DiNP]	Mono(4-methyl-7-carboxyheptyl) phthalate [7cx-MMeHP]	0.02	Yes	12	2
	Mono(4-methyl-7-hydroxyloctyl) phthalate [7OH-MMeOP]	0.01	No		
	Mono(4-methyl-7-oxooctyl) phthalate [7oxo-MMeOP]	0.02	No		
Perfluorooctanesulfonic acid [PFOS]		0.20	Yes	11	6
Nicotine	Cotinine	0.20	Yes	9	15

Abbreviations:
[LOD] Limit of detection at three times the standard deviation of the responses in chemical blanks
[CV] Coefficient of variation (%)
[Level] Pollutant concentration (ng/ml) of CV determination

Table S2.

Summary of the technical parameters used in the LC/MS/MS analysis of phthalate metabolites, cotinine, and PFOS in human amniotic fluid. The declustering potentials (Dp) and collision energies (Ce) in volts (V) are tabulated for each analyte and selected reaction monitoring (SRM) transition

Analyte	SRM transition (m/z)	Dp (V)	Ce (V)
5cx-MEPP	307.2→159.2	-45	-19
7cx-MMeHP	321.1→173.1	-45	-23
Cotinine	177.0→80.1	40	30
PFOS	499.0→99.0	-80	-110
7OH-MMeOP	307.2→121.1	-45	-24
5oxo-MEHP	291.2→121.1	-45	-27
7oxo-MMeOP	305.2→121.0	-45	-23
5OH-MEHP	293.2→121.1	-45	-22
[$^{2}H_4$]-5cx-MEPP	311.2→159.2	-45	-19
[$^{2}H_4$]-7cx-MMeHP	325.2→173.0	-45	-23
[$^{2}H_3$]-Cotinine	180.0→80.1	40	30
[$^{13}C_4$]-PFOS	503.0→80.0	-80	-110

Abbreviations: [5cx-MEPP] mono(2-ethyl-5-carboxypentyl) phthalate, [7cx-MMeHP] mono(4-methyl-7-carboxyheptyl) phthalate, [PFOS] perfluorooctanesulfonic acid, [7OH-MMeOP] mono(4-methyl-7-hydroxyoctyl) phthalate, [5oxo-MEHP] mono(2-ethyl-5-oxohexyl) phthalate, [7oxo-MMeOP] mono(4-methyl-7-oxooctyl) phthalate, [5OH-MEHP] mono(2-ethyl-5-hydroxyhexyl) phthalate

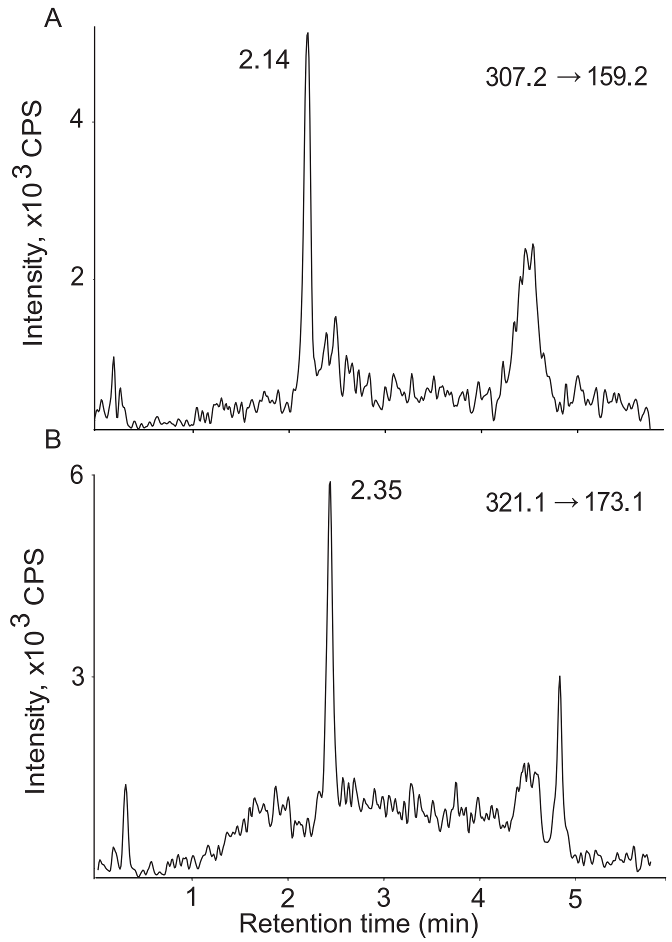

Figure S1. LC/MS/MS chromatograms showing (A) an amniotic fluid sample with 0.2 ng/ml of mono(2-ethyl-5-carboxypentyl) phthalate, (B) an amniotic fluid sample with 0.2 ng/ml of mono(4-methyl-7-carboxyheptyl) phthalate. CPS: Counts per second

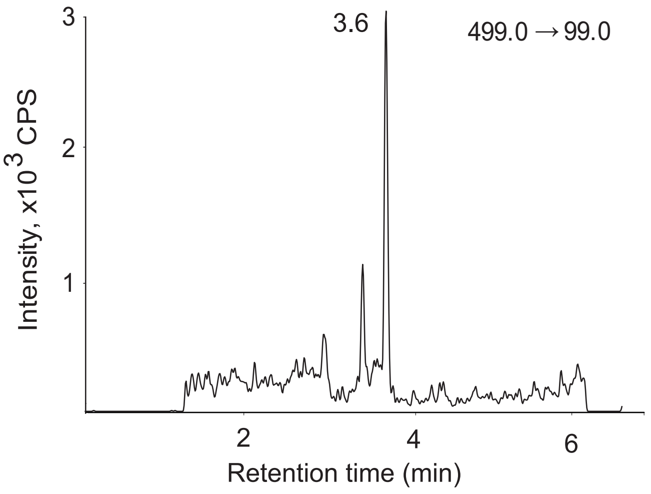

Figure S2. LC/MS/MS chromatogram showing an amniotic fluid sample with 0.2 ng/ml of PFOS.
CPS: Counts per second

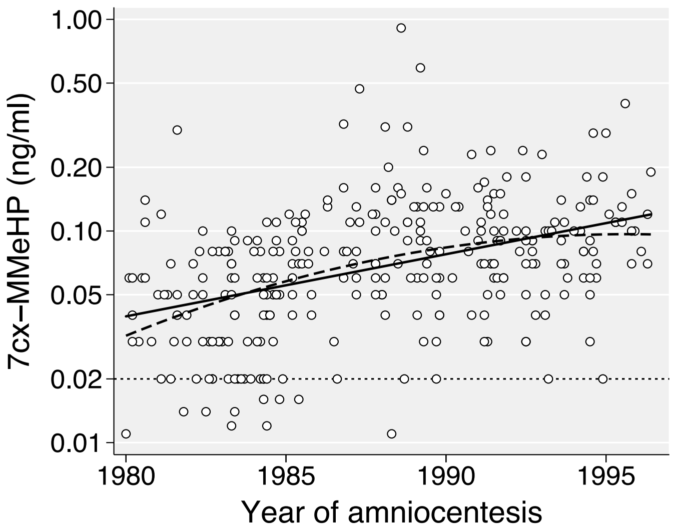

Figure S3. Amniotic fluid mono(4-methyl-7-carboxyheptyl) phthalate [7cx-MMeHP] according to year of amniocentesis. Solid line is unadjusted linear regression. Dashed line is unadjusted linear regression with a squared term of year of amniocentesis showing non-linearity (P= 0.034, testing the null hypothesis of no curvature). Dotted line is limit of detection

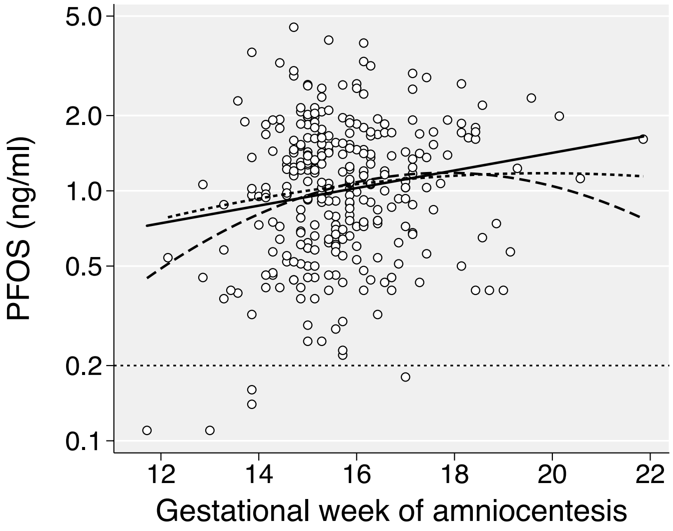

Figure S4. Amniotic fluid perfluorooctanesulfonic acid [PFOS] according to gestational week of amniocentesis. Solid line is unadjusted linear regression. Dashed line is unadjusted linear regression with a squared term of gestational week of amniocentesis showing non-linearity (N= 300, p= 0.008, testing the null hypothesis of no curvature). Short-dashed line is unadjusted linear regression with a squared term of gestational week of amniocentesis and excluding five imputed values below the limit of detection (dotted line) showing non-significant non-linearity (N= 295, p= 0.31, testing the null hypothesis of no curvature)
